# Complete Genome Sequence of *Kosakonia* sp. Strain CCTCC M2018092, a Fucose-Rich Exopolysaccharide Producer

**DOI:** 10.1128/MRA.00567-19

**Published:** 2019-07-25

**Authors:** Songfeng Niu, Wei Ma, Mingyi Jin, Jie Chen, Shanshan Li, Xiang Zou

**Affiliations:** a College of Pharmaceutical Sciences, Southwest University, Chongqing, People’s Republic of China; b Wuhan Sunhy Biology Co., Ltd., Wuhan, People’s Republic of China; c School of Chemical Engineering & Pharmacy, Wuhan Institute of Technology, Wuhan, People’s Republic of China; Georgia Institute of Technology

## Abstract

Kosakonia sp. strain CCTCC M2018092 is a fucose-rich exopolysaccharide producer that was isolated from spring water in Chongqing, Southwest China. In this study, the whole-genome sequence and genetic characteristics of this strain were elucidated. This genome contained 4,789,478 bp, with a G+C content of 56.08% (excluding the plasmid). The genome information in this study will facilitate understanding of the mechanism of high yield of fucose-rich exopolysaccharide produced by *Kosakonia* sp. CCTCC M2018092.

## Abstract (precis)

Kosakonia sp. strain CCTCC M2018092 is a fucose-rich exopolysaccharide producer that was isolated from spring water in Chongqing, Southwest China. In this study, the whole-genome sequence and genetic characteristics of this strain were elucidated. This genome contained 4,789,478 bp, with a G+C content of 56.08% (excluding the plasmid). The genome information in this study will facilitate understanding of the mechanism of high yield of fucose-rich exopolysaccharide produced by *Kosakonia* sp.

## ANNOUNCEMENT

*Kosakonia* sp. strain CCTCC M2018092 was determined to be a Gram-negative, facultatively anaerobic bacterium with a rod cell shape. Most *Kosakonia* species are isolated from soil or trees, and several of them can promote plant growth. However, some other *Kosakonia* species, such as Kosakonia cowanii, are associated with human beings (1, 2). To date, several genera of bacteria have been reported to produce fucose-containing exopolysaccharides, while no literature has reported the production of fucose-containing polysaccharides by *Kosakonia* strains. Fucose-containing oligosaccharides and polysaccharides currently show enormous potential in foods, cosmetics, and pharmaceuticals because of the notable properties of l-fucose, a rare and functional sugar. It has been reported that approximately 50% of human milk oligosaccharides are fucosylated (3) and exhibit efficient prebiotic (4), bacteriostatic (5), and antifungal (6) properties.

*Kosakonia* sp. strain CCTCC M2018092 was cultured aerobically on Sabouraud medium at 30°C. Genomic DNA was extracted using a TIANamp bacterial DNA kit (Tiangen Biotech., Beijing, China). Whole-genome sequencing of strain CCTCC M2018092 was carried out on PacBio RS and Illumina sequencing platforms (Biozeron Biotechnology Co., Ltd., Shanghai, China). A TruSeq DNA sample prep kit and a TruSeq sequencing by synthesis (SBS) kit v3-HS (200 cycles) were used for library construction and DNA sequencing (Illumina HiSeq platform), respectively. The genomic DNA was disrupted into 8- to 10-kbp fragments by the g-TUBE method, followed by PacBio RS platform sequencing on two single-molecule real-time (SMRT) cells. After adapter trimming, 40,025 total reads from PacBio data and 18,637,752 total reads from Illumina data, with 548-fold average coverage, were obtained. The FastQC tool was used to assess the read quality (7). Illumina data were used to evaluate the complexity of the genome and correct the PacBio long reads. ABySS (http://www.bcgsc.ca/platform/bioinfo/software/abyss) was used to perform genome assembly with multiple kmer parameters in order to obtain optimal assembly results (8). Then, Canu (https://github.com/marbl/canu) was used to assemble the corrected PacBio long reads. The GapCloser software (https://sourceforge.net/projects/soapdenovo2/files/GapCloser/) was subsequently applied to fill the remaining local inner gaps and correct the single-base polymorphisms for the final assembly results (9). The whole genome of strain CCTCC M2018092 was annotated using the NCBI Prokaryotic Genome Annotation Pipeline (PGAP) (10). Protein-coding sequences were annotated with a BLASTp search against the NCBI database (https://www.ncbi.nlm.nih.gov/), Kyoto Encyclopedia of Genes and Genomes (KEGG) database (http://www.genome.jp/kegg/), and Clusters of Orthologous Groups (COG) of proteins database (https://www.ncbi.nlm.nih.gov/COG/). All software programs were used with default parameters, unless otherwise stated.

**FIG 1:**
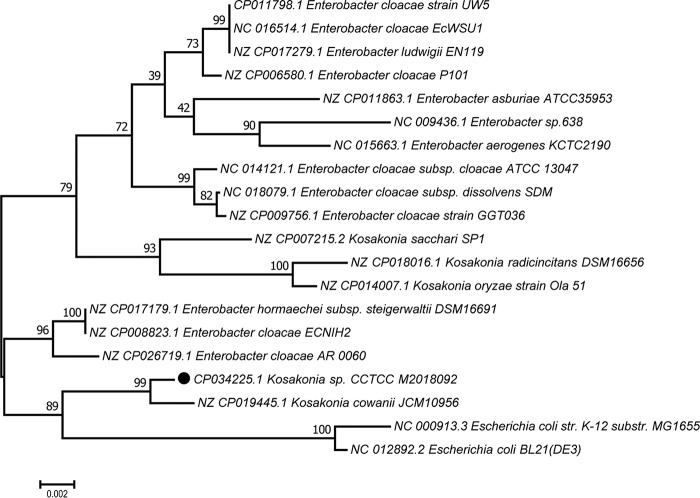
Phylogenetic relationships of the strain CCTCC M2018092. The scale bar represents the distance corresponding to 0.002 changes per nucleotide position. str., strain; substr., substrain.

The 16S rRNA genes were used to construct a phylogenetic tree with the MEGA 7.0 software (11). The neighbor-joining method and 1,000 bootstrap replicates were performed to assess the evolutionary relationships between different strains (Fig. 1). The result showed that *Kosakonia* sp. CCTCC M2018092 is closest to Kosakonia cowanii JCM10956 (GenBank accession number CP019445), and the two of them are classified in the same branch. Furthermore, the two strains are far from the other strains of the genus *Kosakonia* in classification, which indicates that they may not belong to the genus *Kosakonia*.

### Data availability.

The whole-genome sequence has been deposited in the NCBI GenBank database under accession number CP034225. The raw data were submitted to the SRA under accession number PRJNA506809.

## References

[1] Brady C, Cleenwerck I, Venter S, Coutinho T, De Vos P 2013 Taxonomic evaluation of the genus *Enterobacter* based on multilocus sequence analysis (MLSA): proposal to reclassify *E. nimipressuraliss* and *E. amnigenus* into *Lelliottia* gen. nov. as *Lelliottia nimipressuralis* comb. nov. and *Lelliottia amnigena* comb. nov., respectively, *E. gergoviae* and *E. pyrinus* into *Pluralibacter* gen. nov. as *Pluralibacter gergoviae* comb. nov. and *Pluralibacter pyrinus* comb. nov., respectively, *E. cowanii*, *E. radicincitans*, *E. oryzae* and *E. arachidis* into *Kosakonia* gen. nov. as *Kosakonia cowanii* comb. nov., *Kosakonia radicincitans* comb. nov., *Kosakonia oryzae* comb. nov. and *Kosakonia arachidis* comb. nov., respectively, and *E. turicensis*, *E. helveticus* and *E. pulveris* into *Cronobacter* as *Cronobacter zurichensis* nom. nov., *Cronobacter helveticus* comb. nov. and *Cronobacter pulveris* comb. nov., respectively, and emended description of the genera *Enterobacter* and *Cronobacter*. Syst Appl Microbiol 36:309–319. doi:10.1016/j.syapm.2013.03.005. 23632228

[2] Yang X-J, Wang S, Cao J-M, Hou J-H 2018 Complete genome sequence of human pathogen *Kosakonia cowanii* type strain 888-76^T^. Braz J Microbiol 49:16–17. doi:10.1016/j.bjm.2017.03.010. 28774637 PMC5790570

[3] Petschacher B, Nidetzky B 2016 Biotechnological production of fucosylated human milk oligosaccharides: prokaryotic fucosyltransferases and their use in biocatalytic cascades or whole cell conversion systems. J Biotechnol 235:61–83. doi:10.1016/j.jbiotec.2016.03.052. 27046065

[4] Yu Z-T, Chen C, Kling DE, Liu B, McCoy JM, Merighi M, Heidtman M, Newburg DS 2013 The principal fucosylated oligosaccharides of human milk exhibit prebiotic properties on cultured infant microbiota. Glycobiology 23:169–177. doi:10.1093/glycob/cws138. 23028202 PMC3531294

[5] Bode L 2012 Human milk oligosaccharides: every baby needs a sugar mama. Glycobiology 22:1147–1162. doi:10.1093/glycob/cws074. 22513036 PMC3406618

[6] Gonia S, Tuepker M, Heisel T, Autran C, Bode L, Gale CA 2015 Human milk oligosaccharides inhibit *Candida albicans* invasion of human premature intestinal epithelial cells. J Nutr 145:1992–1998. doi:10.3945/jn.115.214940. 26180242

[7] Andrews S 2010 FastQC: a quality control tool for high throughput sequence data. http://www.bioinformatics.babraham.ac.uk/projects/fastqc.

[8] Simpson JT, Wong K, Jackman SD, Schein JE, Jones SJM, Birol I 2009 ABySS: a parallel assembler for short read sequence data. Genome Res 19:1117–1123. doi:10.1101/gr.089532.108. 19251739 PMC2694472

[9] Luo R, Liu B, Xie Y, Li Z, Huang W, Yuan J, He G, Chen Y, Pan Q, Liu Y, Tang J, Wu G, Zhang H, Shi Y, Liu Y, Yu C, Wang B, Lu Y, Han C, Cheung DW, Yiu S-M, Peng S, Zhu X, Liu G, Liao X, Li Y, Yang H, Wang J, Lam T-W, Wang J 2012 SOAPdenovo2: an empirically improved memory-efficient short-read *de novo* assembler. Gigascience 1:18. doi:10.1186/2047-217X-1-18. 23587118 PMC3626529

[10] Tatusova T, DiCuccio M, Badretdin A, Chetvernin V, Nawrocki EP, Zaslavsky L, Lomsadze A, Pruitt K, Borodovsky M, Ostell J 2016 NCBI Prokaryotic Genome Annotation Pipeline. Nucleic Acids Res 44:6614–6624. doi:10.1093/nar/gkw569. 27342282 PMC5001611

[11] Kumar S, Stecher G, Tamura K 2016 MEGA7: Molecular Evolutionary Genetics Analysis version 7.0 for bigger datasets. Mol Biol Evol 33:1870–1874. doi:10.1093/molbev/msw054. 27004904 PMC8210823

